# Absence of MCP-induced Protein 1 Enhances Blood–Brain Barrier Breakdown after Experimental Stroke in Mice

**DOI:** 10.3390/ijms20133214

**Published:** 2019-06-30

**Authors:** Zhuqing Jin, Jian Liang, Jiaqi Li, Pappachan E. Kolattukudy

**Affiliations:** 1 School of Basic Medicine, Zhejiang Chinese Medical University, Hangzhou 310053, Zhejiang, China; 2 Burnett School of Biomedical Sciences, University of Central Florida College of Medicine, 4000 Central Florida Blvd, Orlando, FL 32816, USA; 3 School of Pharmacy, Macau University of Science and Technology, Taipa, Macau, China

**Keywords:** Middle cerebral artery occlusion, MCPIP1, blood–brain barrier, inflammatory response, metalloproteinases, tight junction proteins

## Abstract

Focal cerebral ischemia can cause blood–brain barrier (BBB) breakdown, which is implicated in neuroinflammation and progression of brain damage. Monocyte chemotactic protein 1–induced protein 1 (MCPIP1) is a newly identified zinc-finger protein that negatively regulates inflammatory signaling pathways. We aimed to evaluate the impact of genetic MCPIP1 deletion on BBB breakdown and expression of BBB-related matrix metalloproteinases (MMPs) and tight junction proteins after cerebral ischemia/reperfusion (I/R) using MCPIP1-deficient (MCPIP1^–/–^) mice. Transient middle cerebral artery occlusion was induced in the MCPIP1^–/–^ mice and their wild-type littermates for 2 h followed by reperfusion for 24 h. The degree of BBB breakdown was evaluated by injection of fluorescein isothiocyanate (FITC)-dextran. Quantitative real-time polymerase chain reaction, western blot, and immunohistochemistry were performed to compare the expression of MMPs and claudin-5 and zonula occludens-1 (ZO-1). MCPIP1 deficiency in mice resulted in enhanced leakage of FITC-dextran, increased expression of MMP-9/3, and reduced expression of claudin-5 and ZO-1 in the brain compared to that seen in their wild-type littermates subjected to cerebral I/R. These results demonstrate that absence of MCPIP1 exacerbates cerebral I/R-induced BBB disruption by enhancing the expression of MMP-9/3 and the degradation of claudin-5 and ZO-1, providing novel insights into the mechanisms underlying BBB breakdown after cerebral ischemia/reperfusion

## 1. Introduction

Ischemic stroke is a devastating neurological disorder with high mortality and limited functional recovery [1]. Blood–brain barrier (BBB) breakdown is a common consequence of stroke, contributing to further progression of cerebral ischemic injury [2,3]. BBB homeostasis relies mainly on interactions among endothelial cells, astrocytes, and extracellular matrix [4]. Inflammatory signaling activated by ischemic stroke is a critical event in BBB breakdown during ischemic stroke [5,6]. Matrix metalloproteinases (MMPs), such as MMP-9/3 activated by upregulation of pro-inflammatory cytokines (i.e., TNF-α, IL-1β), are known to breakdown of the extracellular matrix and disruption of the BBB in many experimental stroke models [5,7,8]. Following stroke, activation of MMPs can also degrade tight junction proteins claudin-5 and zonula occludens (ZO)-1 [9,10], leading to loss of BBB tight junction integrity, which results in increased paracellular permeability, vasogenic edema, and increased mortality. Both claudin-5 and ZO-1 have been shown to be the essential integral proteins in tight junction, and their levels have been considered as indicators of normal and dysfunctional states of the BBB [4].

Monocyte chemoattractant protein-1 (MCP-1) is a key chemokine expressed in astrocytes, endothelial cells, neurons, and microglia in response to ischemic stroke [11,12]. MCP-1 has been shown to increase leakage of fluorescin isothiocyanate (FITC)-albumin into the brain [11,13]. Mice lacking MCP-1 displays reduced leukocyte infiltration and reduced production of inflammatory cytokines (i.e., TNF-α, IL-1β, IL-6), with a smaller size of infarct after transient focal ischemia [12,14]. We recently reported that MCP-1 binding to its receptor CCR2 induces a novel zinc-finger protein, named MCP-1-induced protein 1 (MCPIP1) [15], which was further identified as a negative regulator of inflammatory response by inhibition of NF-κB signaling pathway [16]. MCPIP1 also functions as a RNase promoting inflammatory mRNA degradation [17]. MCPIP1-deficient mice develop spontaneously systemic inflammation with elevated circulating inflammatory cytokines [16,17]. These mice also showed the upregulation of proinflammatory cytokines in the brain and an increase of infarct volume in a mouse model of ischemic stroke [18,19]. This novel finding may be of great importance to the pathogenesis of ischemic stroke, where the degree of MCPIP1 expression may influence the degree and extent of inflammation and/or BBB breakdown. Therefore, we investigated the impact of MCPIP1 deficiency on BBB homeostasis in a well-defined murine model of stroke. As we demonstrate in this report, MCPIP1-deficient mice displayed an increased leakage of FITC-dextran into the cortical tissue compared to their wild-type littermates following transient focal ischemia. MCPIP1-deficient mice also showed alterations in expression patterns of MMP-9/3, claudin-5 and ZO-1 proteins, which predispose them to an extremely high susceptibility to BBB breakdown. These results strongly suggest that MCPIP1 plays an important role in maintaining the integrity of BBB after ischemic stroke and regulate the extent of the BBB leakage.

## 2. Results

### 2.1. MCPIP1 Is Up-Regulated in the Murine Brain Subjected to Transient Focal Ischemia/Reperfusion (I/R) Injury

**Figure 1:**
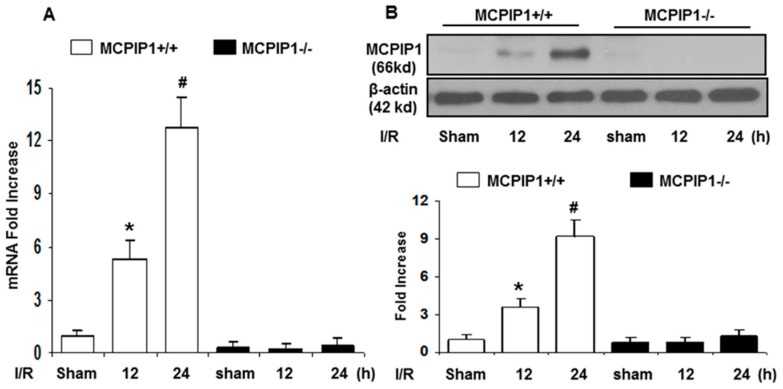
Monocyte chemotactic protein 1–induced protein 1 (MCPIP1) mRNA and protein levels are elevated in the murine brain subjected to ischemia/reperfusion (I/R) injury. (**A**) MCPIP1 mRNA expression in the mouse brain with I/R insult was measured by qRT-PCR. Values represent mean ± SD, *p < 0.05, # *p* < 0.01 versus sham-operated controls; *n* = 6 per group. (**B**) MCPIP1 protein levels in mouse brain with I/R insult were measured by western blot. Results are representative of three independent experiments. Values represent mean ± SD, * *p* < 0.05, # *p* < 0.01 versus sham-operated controls; *n* = 6 per group.

To examine the potential role of MCPIP1 in ischemic stroke, we tested whether MCPIP1 is induced in the murine brain after I/R injury. As shown in Figure 1, MCPIP1 expression at transcript levels in the ipsilateral side of the brain cortex of the MCPIP^+/+^ mice subjected to I/R were significantly elevated compared to that of the sham-operated controls. MCPIP1 mRNA was up-regulated at 12 h after I/R, and the transcript levels reached 12.8 ± 1.6-fold 24 h after I/R injury in the MCPIP^+/+^ mice (Figure 1A). Consistently, elevated MCPIP1 proteins were documented by western blot analysis. The 66 kDa of MCPIP1 was detected at 12 h after I/R insult and its expression continuously increased up to 9.8 ± 1.3 fold at 24 h after I/R (Figure 1B). MCPIP1 was not detected at both transcript and protein levels in MCPIP1^–/–^ mice subjected to I/R or the sham-operated controls at 24 h of I/R injury (Figure 1A,B). These results indicated that high levels of MCPIP1 were induced in the wild-type mice, but not in the MCPIP1^–/–^ mice subjected to focal ischemia/reperfusion injury.

### 2.2. Extravasation of FITC-Dextran Is Markedly Increased in the Brains of MCPIP1^–/–^ Mice Subjected to Transient Focal I/R Injury

**Figure 2:**
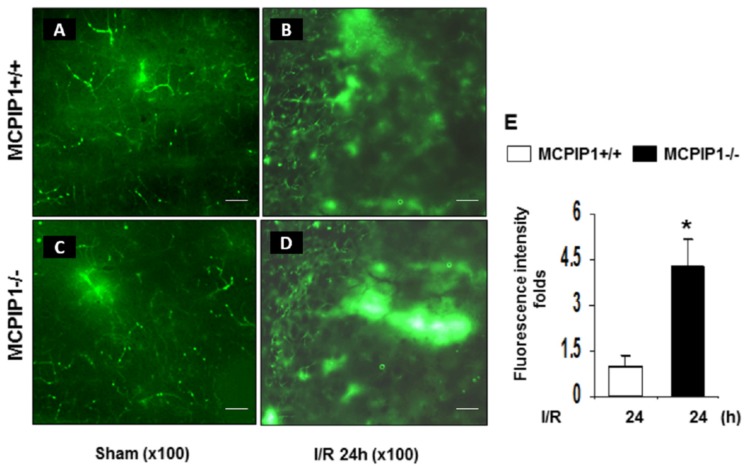
MCPIP1 deficiency exacerbates extravasation of fluorescein isothiocyanate (FITC)-dextran in the injured regions of the brains after 24 h reperfusion following MCAO. (**A**–**D**) Representative images of FITC-dextran extravasation in the brains from the MCPIP1^–/–^ mice and their wild-type littermates underwent 2 h MCAO followed by 24 h reperfusion. (**E**) Measurement of fluorescence intensity in the ischemic tissue. The MCPIP1^–/–^ mice had a significant increase in the leakage of FITC-dextran compared to their wild-type littermates after 2 h of MCAO and 24 h of reperfusion. Values represent mean ± SD, * *p* < 0.05 versus wild-type group. *n* = 6 per group. Scale bar, 25 µm.

BBB disruption is strongly implicated in the pathogenesis of acute ischemic stroke and plays a key role in the evolution of infarction volume [2,3]. Since MCPIP1^–/–^ mice had an increased infarct volume compared to their wild-type littermates after reperfusion following middle cerebral artery occlusion (MCAO) [18,19], we examined if BBB permeability is increased after reperfusion following MCAO in MCPIP^–/–^ mice. BBB disruption in MCPIP1^–/–^ mice was assessed in vivo using FITC-dextran by fluorescent immunohistochemistry analysis. As shown in Figure 2, transient focal ischemia by MCAO following by 24 h of reperfusion caused circulating FITC-dextran extravasating into the peri-infarct cortex of the brain compared to the sham-operated controls in both the MCPIP1^–/–^ mice and their wild-type littermates (Figure 2A). Measurement of fluorescence intensity in peri-infarct cortical regions of the I/R hemisphere showed that the leakage of FITC-dextran was markedly increased up to 4.2-fold in the injured cortex of the MCPIP1^–/–^ mice compared to the leakage seen in their wild-type littermates after 24 h reperfusion following MCAO (Figure 2B), indicating that BBB disruption is markedly increased in the MCPIP1^–/–^ mice subjected to transient focal I/R injury. 

### 2.3. Matrix Metalloproteinase Expression in MCPIP1^–/–^Mice Subjected to Transient Focal I/R Injury

**Figure 3:**
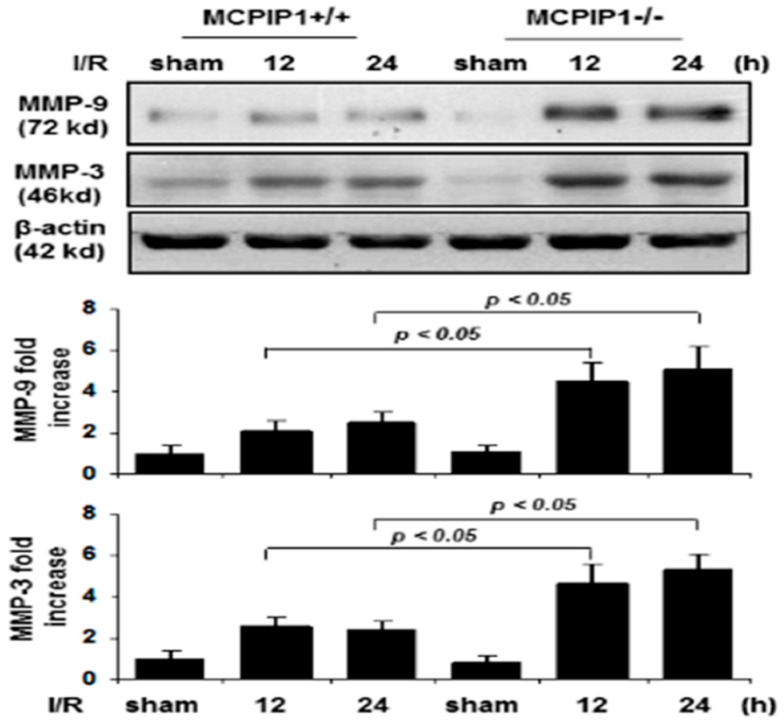
Altered expression of matrix metalloproteinase MMP-9/3 in MCPIP1^–/–^ mice subjected to transient focal I/R injury. Representative immunoblots analysis of MMP-9/3 protein levels in the mice brains after 2 h of MCAO following by 12 or 24 h of reperfusion. Protein extracts from the mice brains were subjected to immunoblot analysis with anti-MMP-9 or anti-MMP-3 antibodies, and β-actin served as a loading control (*Upper panels*). The bar graphs show the densitometric analysis of levels of MMP-9 (*Middle panel*) and MMP-3 (*Lower panel*) normalized by β-actin and expressed as fold change compared the sham-operated animals. Expression of MMP-9/3 was significantly higher in MCPIP1^–/–^ mice compared to that seen in their wild-type littermates subjected to reperfusion for 12 or 24 h after MCAO. Values represent mean ± SD, * *p* < 0.05 versus wild-type littermates. *n* = 6 per group.

Cerebral ischemia-induced up-regulation of inflammatory cytokines such as TNFα and IL-1β are known to stimulate the expression of MMPs that can digest tight junction and basement membrane proteins, and thus contribute to BBB disruption [20]. We examined whether the increased FITC-dextran leakage in the MCPIP1^–/–^ mice is associated with the expression of MMP-3/9 in the brains after transient focal I/R injury. As shown in Figure 3, the expression of MMP-3/9 in mice lacking MCPIP1 was significantly increased at 12 and 24 h of reperfusion following 2 h of MCAO compared to that seen in the sham-operated controls and their wild-type littermates, assessed by immunoblots. 

### 2.4. Altered Expression of Tight Junctions in MCPIP1^−/−^ Mice Subjected to Transient Focal I/R Injury

**Figure 4:**
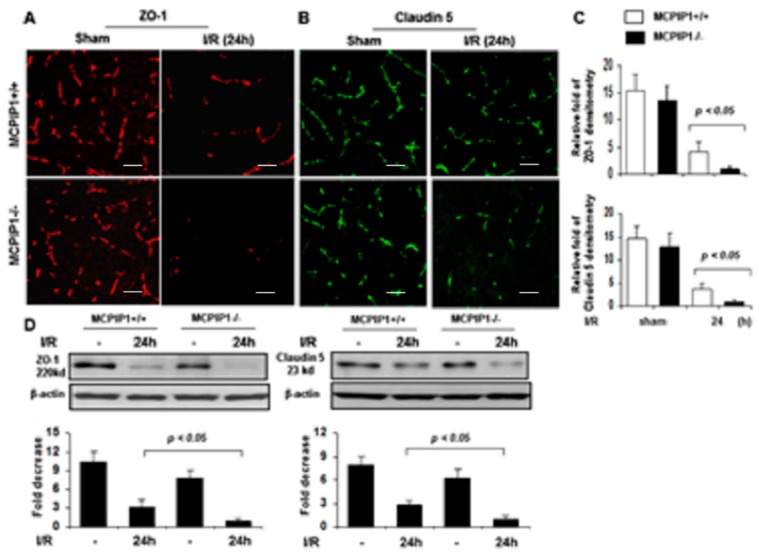
Altered expression of tight junction proteins zonula occludens-1 (ZO-1) and claudin-5 in the brains of MCPIP^–/–^ mice subjected to transient focal ischemia/reperfusion. (**A**,**B**) Representative photomicrographs of immunofluorescence staining using AlexaFluor^®^-conjugated secondary antibodies. The red fluorescence indicates expression of ZO-1 and the green fluorescence indicates expression of claudin-5 in brain sections of the MCPIP1^–/–^ mice and their wild-type littermates subjected to 2 h of MCAO followed by 24 h of reperfusion. The relative levels of ZO-1 and claudin-5, assessed by immunofluorescence intensity, are presented in bar graphs (**C**). Levels of ZO-1 and claudin-5 were significantly reduced in the MCPIP1^–/–^ mice compared to that seen in their wild-type littermates subjected to 24 h of reperfusion after 2 h of MCAO. Values represent mean ± SD. *p* < 0.05, *n* = 6 per group. (**D**) Protein extracts from the mice brains were subjected to immunoblot analysis with anti-ZO-1 or anti-claudin 5 antibodies, and β-actin served as a loading control (*Upper panels*). Bar graphs show the levels of ZO-1 (*Lower panel, left*) and claudin-5 (*Lower panel, right*), assessed by densitometric analysis normalized by β-actin protein levels and expressed as fold change compared the sham-operated animals. Levels of ZO-1 and claudin-5 in the brain of the MCPIP1^–/–^ mice were significantly reduced compared to that seen in their wild-type littermates subjected to 24 h of reperfusion after MCAO I/R hemisphere was measured by western blot with mixed samples of three independent experiments. Values represent mean ± SD. *p* < 0.05 between the MCPIP1^–/–^ mice and their wild-type littermates. *n* = 6 per group. Scale bar, 25 µm.

The BBB is mainly formed by specialized brain endothelial cells that are held together by well-developed tight junctions to keep its structure integrity [4]. Given that MCPIP^–/–^ mice showed an increase in the leakage of FITC-dextran in the brains, we examined whether absence of MCPIP1 impairs protein expression of tight junction components in brains of MCPIP1^–/–^ mice by immunostaining with antibodies against tight junction proteins claudin-5 and ZO-1. As shown in Figure 4, expression of tight junction proteins ZO-1 and claudin-5, two important integral membrane elements responsible for the endothelial–endothelial junction, were significantly reduced in the brains of both MCPIP1^–/–^ mice and their wild-type littermates after 24 h reperfusion following 2 h of MCAO compared to the sham-operated controls (Figure 4A,B). Measurement of immunofluorescence intensity of ZO-1 and claudin-5 demonstrated that levels of tight junction proteins claudin-5 and ZO-1 were significantly lower in MCPIP1^–/–^ mice compared to their wild-type littermates after 24 h of reperfusion following 2 h of MCAO (Figure 4C), which was further confirmed by the immunoblot analysis (Figure 4D). Thus, the decreased expression of ZO-1 and claudin-5 in the brains of MCPIP1^–/–^ mice is consistent with the notion of an increased leakage of FITC-dextran in MCPIP1^–/–^ mice subjected to transient focal I/R injury. 

## 3. Discussion

We show for the first time that the BBB is markedly impaired in the injured brains of MCPIP1^–/–^ mice subjected to transient focal ischemia/reperfusion injury. Consistent with the significantly increased FITC-dextran leakage into the injured brain, the protein expression of MMP-9/3 was significantly increased and the expression of tight junction proteins ZO-1 and Claudin-5 was severely impaired in the injured brains of MCPIP1^–/–^ mice compared to that seen in their wild-type littermates. More recently, our group has demonstrated that MCPIP1^−/−^ mice develop the increased infarct volume after 24 h of reperfusion following 2 h of MCAO, accompanied by the upregulation of inflammatory cytokines TNF-α, IL-1β, and IL-6 [18,19]. Collectively, these findings suggest that the loss of MCPIP1 has significant consequences in cerebral I/R-induced BBB disruption and brain damage. 

The loss of BBB function in ischemic stroke has been extensively studied [2,3]. Proinflammatory cytokines and chemokines induced by cerebral I/R injury are major mediators that cause BBB disruption and dysfunction, resulting in progression of stroke-related brain damage [5,6,13]. In particular, cytokines, like TNF-α and IL-1β, can trigger the early events that cause BBB breakdown and subsequent development of cerebral injury [5,6]. In previous studies we showed that MCPIP1 is up-regulated in the brain by LPS preconditioning and electroacupuncture, which plays a critical role in ischemic brain tolerance [18,19]. Also, we demonstrated that absence of MCPIP1 significantly increases the infarct volume, accompanied by an increase in the expression of TNF-α, IL-1β, and IL-6 after I/R injury [18,19]. To further characterize the role of MCPIP in BBB functional integrity after cerebral I/R injury, in the present study we intravenously administered FITC-labeled dextran and determined extravasation of FITC-dextran within the injured cortex. We demonstrated that absence of MCPIP1 significantly enhanced FITC-dextran leakage in the MCPIP1^–/–^ mice compared to that seen in their wild-type littermates subjected to I/R injury, suggesting the loss of MCPIP1 activity impairs brain BBB integrity and function.

The structural integrity of BBB is mainly controlled by the extracellular matrix components and tight junction components [7,8,9,10]. Studies have suggested that MMPs degrade the vascular basement membrane components, resulting in disruption of BBB [7,8]. It has been demonstrated that protein and activity levels of MMP-3 and -9 are increased after stroke and MCAO [21,22]. Consistent to these findings, we showed that absence of MCPIP1 caused a significant increase in the expression of MMP-3 and -9 in the brain compared to that seen in their wild-type littermates after I/R injury, suggesting that upregulation in protein levels of MMP-3 and -9 may cause aberrant proteolysis of extracellular matrix components of BBB, thus resulting in BBB disruption and dysfunction in mice lacking MCPIP1.

**Figure 5:**
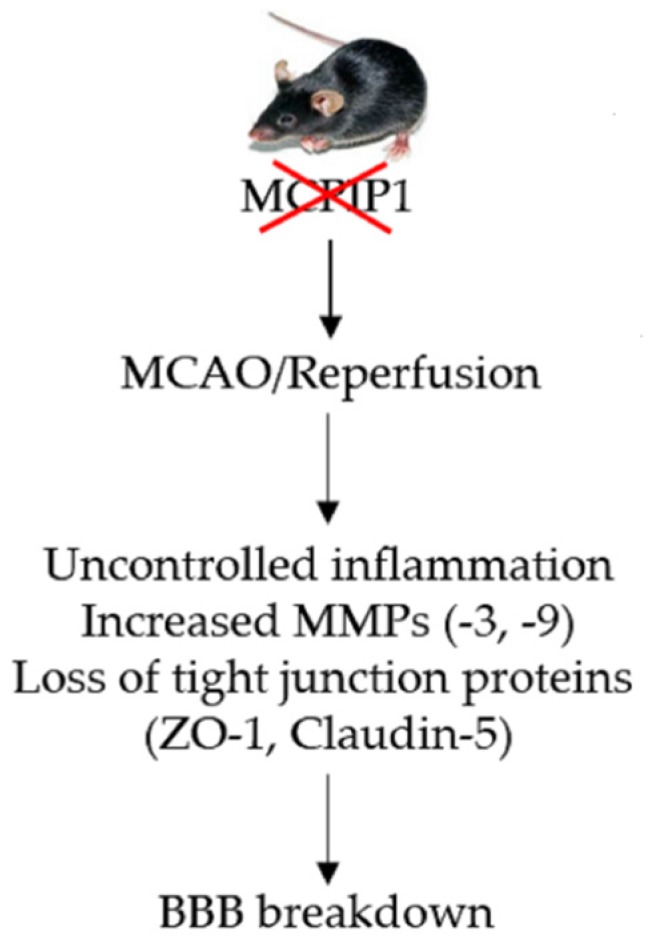
Schematic illustration of MCPIP1 deficiency enhancing blood–brain barrier (BBB) breakdown in mice subjected to middle cerebral artery occlusion (MCAO)/reperfusion injury. We show that deletion of MCPIP1 results in uncontrolled inflammation in the ischemic tissue, which increases the expression of MMP-3/-9 and subsequent degradation of BBB-related tight junction proteins ZO-1 and claudin-5, leading to enhanced BBB breakdown in the MCPIP^–/–^ mice upon ischemia/reperfusion injury.

In addition, we showed that MCPIP1 deficiency leads not only to induction of MMP-3 and -9, furthermore, the expression profile of the tight junction proteins is altered in response to cerebral I/R injury. In contrast to their wild-type littermates, MCPIP1^–/–^ mice developed increased FITC-dextran leakage and reduced expression of tight junction proteins claudin-5 and ZO-1 after 24 h of reperfusion following 2 h of MCAO, suggesting the BBB structural integrity and function are exacerbated by absence of MCPIP1 in mice. Both claudin-5 and ZO-1, two most widely studied components of BBB tight junction proteins, are crucial to maintain BBB homeostasis [23], and they are also vulnerable being degraded by MMPs such as MMP-3 and -9 [24,25]. Since the degradation of claudin-5 and ZO-1 are highly correlated with the dynamic process of BBB break down following cerebral ischemia [9,10,26], the reduced expression of tight junction proteins claudin-5 and ZO-1 in the MCPIP–/– mice after cerebral I/R may contribute to the BBB disruption, leading to enhanced extravasation of FITC-dextran seen in the MCPIP–/– mice (Figure 5). 

## 4. Materials and Methods

### 4.1. Animals

MCPIP1-deficient (MCPIP1^–/–^) mice were produced from the C57BL/6 strain as previously described [16] and maintained in the University of Central Florida Animal Facility. MCPIP1^–/–^ mice were generated by interbreeding of MCPIP1^+/-^ mice and identified by genotyping using ear tissue samples at age of 4–6 weeks. Wild-type littermates (MCPIP^+/+^) were generated through heterozygous mating. Mice were maintained in a 12-h light/dark cycle with a temperature of 22 ± 1 °C and free to access food and water. Male MCPIP1^+/+^ and MCPIP1^−/−^ mice, 8- to 10-week-old, were used for the study. The experimental protocol was approved by the Institutional Animal Care and Use Committee of University of Central Florida (IACUC Number 14-29, approved date: 8 May 2015).

### 4.2. Transient Focal Brain Ischemia/Reperfusion in Mice

To induce transient focal brain ischemia/reperfusion injury, mouse transient middle cerebral artery occlusion (MCAO) was induced as previously described [18,19]. In brief, mice were anesthetized by inhalation of 3% isoflurane and maintained with 1.2% of isoflurane mixed with oxygen-enriched air via a nose cone, and body temperature was maintained at 37 ± 0.5 °C with a heating pad during the procedure. Unilateral MCAO was induced by inserting a 7-0 nylon monofilament into the internal carotid artery via an external carotid artery stump and filament is advanced up to 8–9 mm into the right middle cerebral artery (MCA) from the right common carotid artery junction. After MCAO for 120 min, the occluding filament was gently removed from the MCA to allow reperfusion, and the animals were maintained individually in a heated cage. After completion of the reperfusion period, the mice were euthanized with an overdose of pentobarbital and then transcardially perfused with ice-cold PBS containing heparin, followed by 4% (*w*/*v*) paraformaldehyde in PBS. The mice brains were then removed for further analysis.

### 4.3. Assessment of BBB Breakdown Using FITC-Dextran

Fluorescein isothiocyanate- dextran (FITC-dextran, 500 mg/kg, Sigma-Aldrich, St. Louis, MO, USA) was used to evaluate BBB breakdown as previously described [27]. Briefly, at the end of 24 h reperfusion after 2 h MCAO, FITC-dextran (500 mg/kg, Sigma, USA) was given intravenously to mice over 5 min. The mice were then perfused transcardiacally as described above. The mice brains were removed and fixed in 4% paraformaldehyde in PBS at 4 °C for 24 h. The fixed-brains were then sliced into 50-μm-thick coronal sections with a freezing microtome. Sections were mounted onto microscope glass slides with Vectashield mounting medium (Vector Lab., Burlingame, CA, USA). Multiple fields were visualized under a fluorescence microscope (Leica TCS SP5, Buffalo Grove, IL, USA) to evaluate the FITC-dextran leakage. Three randomly selected fields from each section were captured and six animals were studied per group.

### 4.4. Quantitative Real-Time PCR

Quantitative real time PCR (qRT-PCR) was conducted as we previously described [18,19]. Briefly, total RNA was extracted from the mice brains using TRIzol reagent (Invitrogen, Carlsbad, CA, USA). 1 μg of RNA were reverse transcribed to cDNA using a commercially available kit (Applied Biosystems, USA). qRT-PCR was performed with iCycler Thermal Cycler (Bio-Rad, Hercules, CA, USA) using 2 X SYBR Green master mixes (Bio-Rad, USA). All samples were subjected to forty cycles under the following conditions: 95 °C for 30 s, 60 °C for 30 s, proceeded by 10 min at 95 °C for polymerase activation. Quantification was performed by the delta cycle time method for measurement of MCPIP1 transcript using the mouse MCPIP1 primers (forward 5’-CCCCCTGACGACCCTTTAG-3‘, reverse 5‘-GGCAGTGGTTTCTTACGAAGGA-3‘) and mouse β-actin primers (forward 5‘-AAATCGTGCGTGACATCAAAGA-3‘, reverse 5‘-GGCCATCTCCTGCTCGAA-3‘) were used for normalization of MCPIP1 expression in each sample. The relative gene expressions expressed as the ratio of β-actin expression.

### 4.5. Western Blots

Western blot was performed as we previously reported [18,19]. Briefly, brain samples were homogenized on ice in lysis buffer containing protease inhibitors, and the supernatant was collected after centrifugation. Protein concentrations were measured using the Bradford assay (Bio-Rad, Hercules, CA, USA). Equal amounts of protein were loaded, separated with 10% or 12.5% SDS-PAGE gels, and then transferred from gel to nitrocellulose membranes in transfer buffer containing 0.1% SDS. The membrane were blocked with 5% nonfat dry milk in 0.05% Tween-20 in Tris-buffered saline (TBS) for 2 h to prevent no-specific bindings, then incubated with one of the following antibodies: anti-MMP-3, anti-MMP-9 (Cell Signaling, Danvers, MA, USA), anti-ZO-1, anti-claudin 5, and anti-MCPIP1 (Santa Cruz, Dallas, TX, USA) respectively, gently rocking overnight at 4 °C. After washing, the membranes were incubated with the appropriate horseradish peroxidase-conjugated secondary antibodies (Santa Cruz) for 1 h at room temperature. After three times of washing with TTBS, immunoreactive bands were visualized with SuperSignal West Pico Chemiluminescent Substrate (Pierce, Rockford, lL, USA). The intensity of immunoreactive bands were quantified by AlphaImage 2200 (AlphaInnotech, San Leandro, CA, USA) and normalized with protein loading control visualized with an anti-β-actin antibody (Santa Cruz). 

### 4.6. Immunohistochemistry

The mice brains perfused with 4% paraformaldehyde were post-fixed with 4% paraformaldehyde in 4 °C overnight and sectioned coronally with a vibrating microtome (Leica Microsystems, Wetzlar, Germany). Slides containing 30 μm-thick of serial sections were used for immunostaining. The nonspecific binding sites in the brain tissues were blocked with 3% bovine serum albumin in 0.1% TX-100 in PBS for 1 h at room temperature. After washing, the sections were incubated with primary antibodies against GFAP, CD11b (BD Biosciences Pharmingen, San Diego, CA, USA), NSE (Invitrogen, Carlsbad, CA, USA), CD31, Claudin-5, ZO-1 (Invitrogen, USA), and MCPIP1 (Santa Cruz Biotechnology, Dallas, Texas, USA) overnight at 4 °C, respectively. AlexaFluor^®^-488-conjugated or AlexaFluor^®^-594-conjugated secondary antibodies (Invitrogen, Carlsbad, CA, USA) were applied to the sections and incubated for 1 h at room temperature. After washing, the sections were mounted with Vectashield mounting medium (Vector Laboratories, Burlingame, CA, USA) and visualized under a fluorescence microscope (Leica TCS SP5) equipped with a digital camera. Three randomly selected fields were captured from each section and six animals were studied per group. 

### 4.7. Statistical Analysis

All values are presented as mean ± standard deviation. Data were analyzed by one-way analysis of variance (ANOVA) followed by Tukey–Kramer tests for multiple comparisons. A two-side, *p*-value < 0.05 was considered significant.

## 5. Conclusions

In summary, our results define a novel role of MCPIP1 in BBB damage induced by ischemia/reperfusion in mice. We propose that absence of MCPIP1 causes overproduction of ischemia-induced inflammatory cytokines, resulting in MMP-3/9-medaited degradation of claudin-5 and ZO-1 from the extracellular matrix, leading to ischemic BBB disruption and dysfunction. Our findings in the present study may help to develop a potential therapeutic target to protect the BBB against ischemic damage.

## Abbreviations

I/R	ischemia/reperfusion
MCAO	middle cerebral artery occlusion
MMPs	matrix metalloproteinases
BBB	blood–brain barrier
MCPIP1	monocyte chemotactic protein 1–induced protein 1
FITC	fluorescein isothiocyanate
ZO-1	zonula occludens-1
MCP-1	monocyte chemoattractant protein-1
TNF-α	tumor necrosis factor-alpha
IL-1β	interleukin 1 beta
IL-6	interleukin 6
LPS	lipopolysaccharide
qRT-PCR	quantitative real time PCR
